# Effect of intracameral injection of fibrin tissue sealant on the rabbit anterior segment

**Published:** 2010-06-12

**Authors:** Annabel C.Y. Chew, Donald T.H. Tan, Rebekah Poh, Htoon HM, Roger W. Beuerman, Jodhbir S. Mehta

**Affiliations:** 1 Singapore National Eye Centre, Singapore; 2 Singapore Eye Research Institute, Singapore; 3 Department of Ophthalmology, Yong Loo Lin School of Medicine, National University of Singapore, Singapore; 4 Department of Clinical Sciences, Duke-NUS Graduate Medical School, Singapore; 5 SRP Neurosciences and Behavioral Disorders, Duke-NUS, Singapore

## Abstract

**Purpose:**

To investigate the effect of intracameral injection of fibrin tissue sealant on the anterior segment structures in a rabbit model.

**Methods:**

One eye of 10 rabbits received an intracameral injection of fibrin tissue sealant with a thrombin concentration of 500 IU (TISSEEL), and the fellow eye received an intracameral injection of balanced salt solution as a control. The rabbits were followed up with serial slit-lamp examinations, photography, high resolution anterior segment optical coherence tomography scans with pachymetry measurement, and intraocular pressure (IOP) monitoring until complete dissolution of the fibrin sealant. Corneal endothelial cell viability was evaluated using live/dead cell assays. Apoptosis of the cornea and trabecular meshwork were evaluated using TUNEL assays. Ultra-structural examinations of the cornea and trabecular meshwork were performed using electron microscopy. Histology of the trabecular meshwork and iris were analyzed using light microscopy.

**Results:**

The quantity of the intracameral fibrin sealant was shown to be significantly correlated with increased IOP and pachymetry post-operatively. Complete dissolution of the fibrin sealant occurred between 15 and 30 days. Live/dead cell assays showed no decrease in viability of the corneal endothelium, and TUNEL assays showed no increase in apoptosis of the corneal epithelium, stroma, endothelium, or trabecular meshwork in the eyes with the fibrin sealant. Light and electron microscopy of the anterior segment structures were unremarkable.

**Conclusion:**

The intracameral use of fibrin glue was associated with a transient increase in IOP and pachymetry. However, there was no evidence of toxicity or structural damage to the corneal endothelium, trabecular meshwork, or iris.

## Introduction

Fibrin adhesives are surgical hemostatic agents derived from human plasma that are designed to reproduce the final steps of the physiologic coagulation cascade to produce a stable fibrin clot. These have been commercially available in Europe since the late 1970s, and have a wide range of uses, such as hemostasis, wound healing, suture support, and as a tissue sealant, in many procedures and across many medical disciplines [1]. In the past ten years, fibrin glue has been successfully used in ophthalmology for conjunctival closure in pterygium surgery [2] and trabeculectomy [3], treating leaking blebs following glaucoma surgery [4], treating corneal perforations [5], performing amniontic membrane transplantation [6], and sutureless lamellar keratoplasty [7].

Fibrin sealant is highly biocompatible, completely biodegradable, and has shown good results with little toxicity for ocular surface use. We hypothesized that it may be used safely intracamerally. We recently reported the use of intracameral injections of TISSEEL fibrin sealant (Baxter, Deerfield, IL) as an adjunct in performing tectonic deep anterior lamellar keratoplasty (DALK) in three cases with pre-existing or intraoperative macroperforation [8]. We chose fibrin sealant as it is biodegradable, can be left in situ (as compared to the non-absorbable cyanoacrylate glue), clots to form a fibrin network, hence exposing the perforation without aqueous leakage from the perforation site (as compared to conventional viscoelastic materials), is adhesive, and does not dislodge easily.

Even though our pilot cases were characterized by a lack of post-operative complications, the effect of intracameral injection of fibrin glue on anterior segment structures, such as the corneal endothelium, iris, and trabecular meshwork, has yet to be fully evaluated. Theoretically, the presence of substantial amounts of fibrin within the anterior chamber or within the chamber angle may be associated with complications such as pupillary block glaucoma, raised intraocular pressure (IOP), and increased anterior chamber inflammation. Even though fibrin is a naturally occurring product produced in the anterior chamber following marked inflammation, TISSEEL is not formed naturally by the eye. TISSEEL is a commercially available product that is currently composed of a mixture of human and synthetic products. Previously, the aprotinin and thrombin components were taken from bovine sources. The aim of our study was to investigate the effect of intracameral injection of fibrin tissue sealant (TISSEEL) on the anterior segment structures in a rabbit model.

## Methods

The animal protocol of the study adhered to the Association for Research in Vision and Ophthalmology Statement for the Use of Animals in Ophthalmic Vision and Research, and was approved by the institutional review board and ethics committees of the Singapore National Eye Centre and Singapore Eye Research Institute.

Ten female New Zealand white rabbits weighing between 2 and 2.5 kg were used for this study. Twenty eyes of the ten rabbits were divided equally into two groups. One eye of each rabbit received an intracameral injection of fibrin sealant with a thrombin concentration of 500 IU (TISSEEL), and the fellow eye received an intracameral injection of balanced salt solution (BSS; Santen, Osaka, Japan) as a control.

### Surgical procedures

The rabbits were anesthetized with an intramuscular injection of ketamine (40 mg/kg), xylazine (4 mg/kg), and topical xylocaine before surgery. A wire lid speculum was placed to separate the eyelids. An operating microscope was positioned over the eye undergoing surgery. The anterior chamber was entered in the superotemporal quadrant through a long corneal tunnel using a 30-G needle bent approximately at 60 degrees. After entry, 0.03 ml of aqueous humor was withdrawn from the eye, and 0.3 ml of air was injected into the anterior chamber to achieve a complete air fill to push back the iris and the lens. TISSEEL fibrin sealant components were constituted according to the standard manufacturer’s instructions before surgery and pre-warmed to 37 °C. The dual syringe system with a common plunger (Duploject; Baxter) that ensured the feeding of equal volumes of the two components into a common joining piece, mixed just before contact, was assembled. Sealer protein concentrate and 500 IU of thrombin was used for a rapid setting mixture. A 25-G needle was attached to the end of the Duploject and the system was primed to remove any residual air from the system. Then, 0.03 to 0.05 ml of fibrin sealant was injected intracamerally. The presence of the air fill in the anterior chamber allowed the fibrin sealant to be directed toward the corneal endothelium. The air bubble was removed after 1 min and the anterior chamber was then irrigated with 0.3 ml of BSS. A 25-G needle was used as we were unable to inject the fibrin glue through a smaller gauge needle. The fibrin sealant polymerized within the needle several times and we had to change the needle whenever that happened. The procedure was repeated in the fellow eye, and the fibrin sealant was replaced with a 0.03 ml injection of BSS as a control.

### Clinical evaluation

Daily detailed clinical examinations of anesthetized rabbits were performed pre-operatively and post-operatively. The examinations included a slit lamp examination (FS-3V Zoom Photo Slit Lamp, Nikon, Japan) to check the degree of inflammation, cornea clarity, the resolution of intracameral fibrin sealant, high resolution anterior segment optical coherence tomography (OCT) scans of the cornea and anterior segment (Visante OCT; Carl Zeiss Meditec, Inc., Dublin, CA), and intraocular pressure (IOP) measurements (Tono-pen XL: Medtronic Solan, Jacksonville, FL). Corneal thickness measurements were obtained using the measurement calipers that accompanied the Visante software. The anterior segment quadrantic scans were analyzed using a graphic program (Image J, NIH, Bethesda, MD) and the mean area of intracameral fibrin sealant (the average of four radial scans) was calculated daily. All rabbits received the following topical medications in both eyes during the entire duration of follow-up: neomycin 1 drop 4 times a day, polymyxin B 1 drop 4 times a day, dexamethasone (Maxitrol; Alcon Laboratories, Fort Worth, TX) 1 drop 4 times a day, atropine (1%) 1 drop twice a day, and Vidisic gel (Bausch & Lomb, Rochester, NY), once a day.

The rabbits were followed up until complete dissolution of the fibrin sealant (the duration of follow-up ranged from 15 to 30 days). One rabbit died during the follow-up period due to an unrelated cause and was excluded from the study. The rabbits were sacrificed with an intravenous injection of sodium pentobarbitol (100 mg/kg). The rabbit eyes were enucleated, and the corneas and a 2 mm rim of the sclera were removed with a blade and scissors, and the irises were peeled from the corneas. The corneo-scleral buttons and irises were stored in Optisol-GS solution until further testing.

### Corneal specimens

#### Endothelial staining for live/dead cell assay

A viability/cytotoxicity assay using calcein-AM and ethidium homodimer-1 (EthD-1) (Molecular Probes, Eugene, OR) was used to assess corneal endothelial cell viability. The components of the viability/cytotoxicity assay were first allowed to thaw to room temperature in a dark room. Calcein-AM is cleaved by cellular esterases present within viable cells to form a membrane-impermeable fluorescent green product. Ethidium homodimer-1 is a fluorescent red marker that binds to nucleic acids and only passes through the compromised membrane of non-viable cells. Phosphate buffered saline (PBS; 10 ml) was added to calcein-AM (15 µl) to obtain a solution of 6 µM of calcein-AM, and PBS (10 ml) was added to EthD-1 (30 µl) to obtain a solution of 6 µM of EthD-1. The corneo-scleral buttons were initially stored in Optisol GS at 4 °C after harvesting from the rabbits (for less than 30 min). In preparation for the staining, each corneal button was gently submersed in PBS to remove any residual growth medium and the center 8.5 mm of the cornea was trephined using a Coronet corneal punch (Network Medical Products, North Yorkshire, UK). Each trephined control and corneal button was then placed into an individual well of a 12-well plate. Each of the reagents (50 µl) was then gently placed on the endothelial surface (to minimize staining of the epithelium). The plate was covered in foil and allowed to incubate at room temperature in darkness for 45 min.

At the end of the incubation, the tissue was gently washed with PBS and placed endothelial side up onto a glass slide. Slides were subsequently examined using a Zeiss Axioplan 2 fluorescence microscope (Zeiss, Oberkochen, Germany) using sequential illumination at wavelengths of 500 nm and 625 nm. This illuminated the calcein-AM positive (live) and EthD-1 positive (dead) cells, respectively. The photographs were analyzed using a graphic program (Image J) and the percentage of dead cells per slide was calculated.

#### Histopathology and TUNEL assay

The rabbit corneal specimens were embedded in optimal cutting temperature freezing compound. Six micron thick sections were cut and positioned on poly-lysine coated glass slides and then air dried for 20 min. They were then subjected to hematoxylin and eosin staining by standard techniques or evaluated for apoptotic cell death using a terminal deoxynucleotidyl transferase assay (In Situ Cell Detection Kit, TMR red; Roche Applied Science, Indianapolis, IN) for terminal deoxynucleotidyl transferase assay dUTP nick end labeling (TUNEL), according to the manufacturer’s instructions. Nulcei were counterstained with 4’,6-diamidino-2-phenlindole (DAPI; Molecular Probes, Eugene, OR). Slides were then mounted and subsequently examined using a Zeiss Axioplan 2 fluorescence microscope (Zeiss, Oberkochen, Germany) using sequential illumination at wavelengths of 540 nm and 580 nm. This illuminated the TUNEL-positive cells red and the rest of the DAPI stained cells blue. The photographs were analyzed using a graphic program (Image J) and the percentage of apoptotic positive cells per slide was calculated.

#### Electron microsopy (EM)–scanning EM and transmission EM

The rabbit corneas were excised, bisected, and immediately fixed in cold 2.0% glutaraldehyde, 2% paraformaldehyde, and 0.1 M sodium cocodylate, pH 7.4 (Electron Microscopy Sciences, Washington, PA) overnight at 4 °C. The tissues were then washed in sodium cacodylate buffer and rinsed with distilled water. The tissues were secondarily fixed in 1% osmium tetra-oxide (Electron Microscopy Sciences) and then dehydrated, dried, and mounted on SEM stubs. They were then sputter coated with 10 nm of gold and examined using a SEM (JSM-5600; JEOL, Tokyo, Japan) at 10 kV. Low power (18×) and high power (500×) SEM images were taken. Thirteen micrographs were taken per corneal button, one in the center and one in each clock hour around the button. For TEM, the tissue was trimmed into smaller pieces. These samples were then post-fixed in 1% osmium tetra-oxide and potassium ferrocynaide. After extensive rinsing with sodium cacodylate buffer, tissues were dehydrated in a graded series of ethanol, and embedded in Araldite (Electron Microscopy Sciences). All semi-thin sections of 0.5–1µm thickness were cut with a Reichert-Jung Ultracut E Ultramicrotome (C. Reichert Optische Werke AG, Vienna, Austria), counter-stained with toluidine blue/basic fuchsin stain, and examined using an Axioplan Zeiss Light Microscope (Carl Zeiss, Germany). All ultra-thin sections of 60–80 nm were collected on copper grids, doubled-stained with uranyl acetate and lead citrate for 20 min each, then viewed and photographed using a Philips EM 208S Transmission Electron Microscope (FEI Electron Optics BV, Eindoven, The Netherlands) at 100kv.

### Trabecular meshwork specimens

#### TUNEL assay, light microscopy, and transmission electron microscopy

The rabbit angle structures underwent light microscopy and TEM and were evaluated for apoptotic cell death using a TUNEL assay, as described above.

### Iris specimens

#### Light microscopy

The rabbit irises underwent light microscopy, as described above.

### Statistical analysis

Repeated measure analysis using a general linear model was conducted to analyze the effect of fibrin glue on IOP and pachymetry separately. Regression analysis was done for pachymetry (as a dependent variable) and the amount of calculated intracameral fibrin glue and IOP (as independent variables) at the following time points: post-operative day (POD) 1, 4, 7, 10, and 15. Univariate analysis was done for each independent variable and multivariate analysis was done for independent and dependent variables. Correlation coefficients were calculated at each time point.

The eyes were divided into three different groups (eyes with intracameral TISSEEL with normal IOP, eyes with intracameral TISSEEL with increased IOP, and control eyes). Tests of anterior segment structure viability of the three groups were compared using a Kruskal-Wallis test and statistical software (SPSS 17.0). The level of significance was a p-value of 0.05. Data was reported as mean±standard error of mean (SEM).

## Results

### Surgical procedures

We were able to coat the corneal endothelium with the fibrin sealant in all the rabbit eyes. However, it was difficult to control the thickness of fibrin sealant injected onto the endothelium. In all cases, excessive sealant inadvertently coated the iris, and in some instances, the sealant also extended to occlude parts of the chamber angle and this was left in situ. The fibrin sealant took between 30 s to 1 min to set. In all cases, the air bubble did not migrate behind the iris or cause any pupillary block.

### Clinical evaluation

All eyes showed mild hyperaemia with mild inflammation (cellular activity grade 1+ and flare grade 1+) in the anterior chamber, which settled with 1 drop of topical dexamethasone 4 times a day and resolved within the first few days post-operatively.

**Figure 1:**
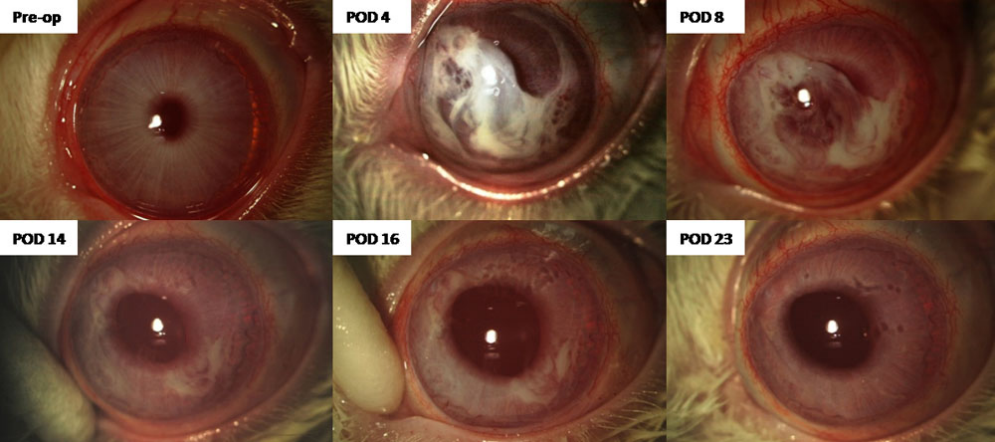
Slit lamp photographs of a rabbit’s eye showing the dissolution of fibrin glue following intracameral injection of fibrin glue into the rabbit’s eye.

**Figure 2:**
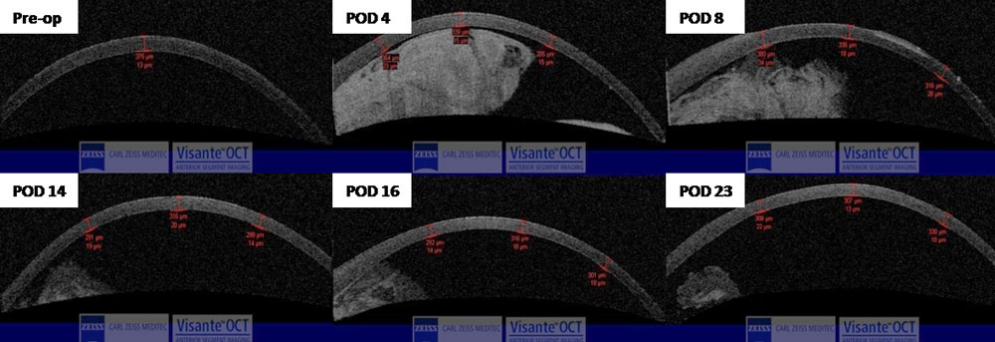
High resolution anterior segment optical coherence tomography showing the effect of the intracameral fibrin glue on the corneal pachymetry of the same rabbit (as in Figure 1). Corneal pachymetry remained thin, between 300 and 370 mm.

**Figure 3:**
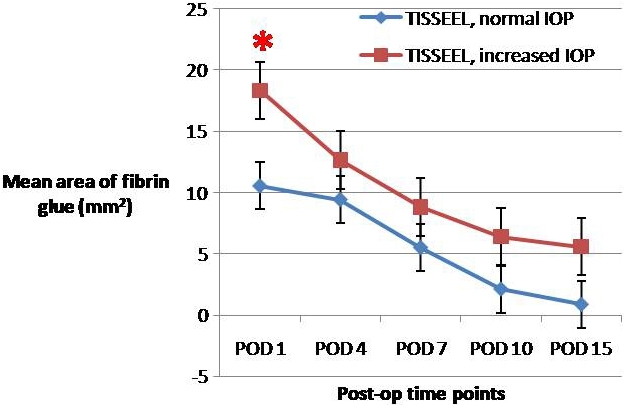
Calculated mean area of intracameral fibrin glue in eyes with increased IOP (n=7) and normal IOP (n=2), showing the rate of dissolution of the intracameral fibrin glue. The average rate of dissolution of TISSEEL in eyes with normal IOP was 0.65 mm^2^/day, while that in eyes with increased IOP was 0.85 mm^2^/day. The asterisk indicates a p<0.05. Error bars are ±SEM.

Of the eyes with the fibrin sealant, two had normal IOP post-operatively, while the other seven eyes had increased IOP. The corneas of the eyes with normal IOP remained thin and clear, despite the presence of fibrin glue coating the endothelium. Of the eyes with increased IOP, all but one showed corneal edema; two eyes had irido-corneal contact with angle closure post-procedure, which resolved spontaneously with the dissolution of the fibrin sealant. The eyes with increased IOP received the following topical anti-glaucoma treatment: 0.5% timolol twice a day, 0.004% travoprost (Travatan; Alcon Laboratories, Fort Worth, TX) once a day, and 0.1% brimonidine tartrate (Alphagan; Allergen, Irvine, CA) three times a day. Six eyes had IOP greater than 25 mmHg, despite intensive topical anti-glaucoma medications, and underwent therapeutic anterior chamber paracentesis with withdrawal of 0.1 ml of aqueous humor. These eyes continued to have IOP spikes of more than 20 mmHg and required anti-glaucoma medication. The IOP returned to normal (less than 20 mmHg) after dissolution of the fibrin glue. In all cases, partial dissolution of the injected fibrin sealant within the anterior chamber was observed to occur within three days after injection, with complete dissolution occurring between PODs 15 and 30. In the control eyes, the IOP and pachymetry remained normal throughout the entire duration of follow-up. Table 1 shows a summary of the clinical details. Figure 1 and Figure 2 show the slit lamp photographs, and ASOCT images of a rabbit. Figure 3 shows the rate of dissolution of the intracameral fibrin sealant.

**Table 1:** Summary of clinical details.

** **	** **	**TISSEEL with normal IOP (n=2)**	**TISSEEL with increased IOP (n=7)**	**Control eyes (n=9)**
Intraocular pressure (mm Hg)	Mean preoperative IOP±SD	13.50±2.12	14.14±2.54	14.44±2.01
	Mean postoperative IOP±SD	12.08±3.31	19.91±8.28	12.49±2.75
Central pachymetry (mm)	Mean preoperative pachymetry±	364.50±14.85	351.14±29.48	347.44±27.78
	Mean postoperative pachymetry±SD	340.88±28.25	759.14±342.03	337.53±27.51

**Figure 4:**
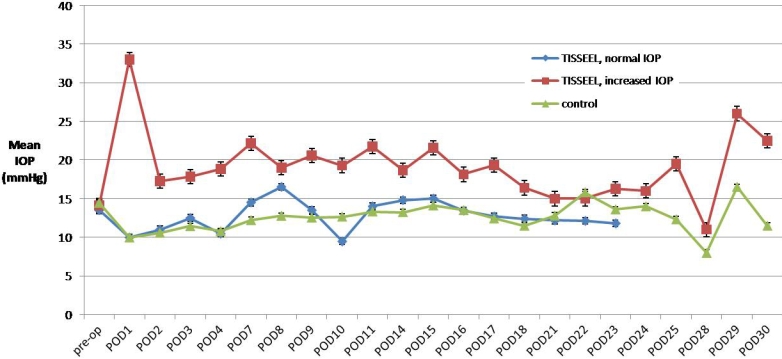
Mean IOP measurements after intracameral injection of fibrin glue in eyes with increased IOP (n=7) and normal IOP (n=2), and control eyes (n=9). The amount of intracameral fibrin sealant was shown to correlate significantly with IOP on POD 1. Error bars are ±SEM.

**Figure 5:**
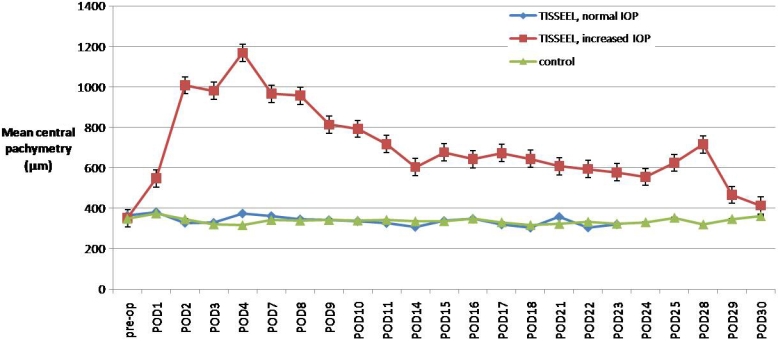
Mean central pachymetry measurements after intracameral injection of fibrin glue in eyes with increased IOP (n=7) and normal IOP (n=2), and control eyes (n=9). Error bars are ±SEM.

The IOP and pachymetry were observed to significantly increase after intracameral injection of the fibrin sealant. Figure 4 and Figure 5 show the mean IOP and central pachymetry of the rabbits in the three different groups. The amount of intracameral fibrin sealant was shown to correlate significantly with IOP on POD 1. There was no correlation between the amount of intracameral fibrin sealant and IOP over other time points, with the p value adjusting for type I errors. The amount of intracameral fibrin sealant was shown to correlate significantly with pachymetry on POD 7 and POD 10, correcting for IOP. There was moderate univariate correlation between increased IOP and increased pachymetry over time, and the correlation was significant. Table 2 shows the univariate analysis correlating the amount of fibrin glue and IOP and Table 3 shows the univariate and multivariate analysis correlating the amount of fibrin sealant, IOP, and pachymetry.

**Table 2:** Univariate analysis of effect of mean area of fibrin glue on IOP, showing significant correlation between area of fibrin glue and IOP on POD 1, p<0.05.

** **	**Univariate analysis correlating area of fibrin glue with IOP**			
IOP (Dependent variable)	R	R square	Significance	Bonferroni adjustment for type I error
POD 1	0.736	0.542	0	0
POD 4	0.257	0.066	0.303	1
POD 7	0.524	0.274	0.026	0.26
POD 10	0.449	0.201	0.062	0.62
POD 15	0.433	0.187	0.073	0.73

There was no correlation between the area of fibrin glue and IOP over other time points, with P value adjusting for type I error.

**Table 3:** Univariate analysis of effects of mean area of fibrin glue on pachymetry, and IOP on pachymetry, showing moderate significant correlation between IOP and pachymetry over time, p<0.05.

** **	** **	**Univariate analysis correlating area of fibrin glue with pachymetry, and IOP with pachymetry**			**Multivariate analysis correlating area of fibrin glue, IOP and pachymetry**		
		R	R square	Significance	R	R square	Significance
POD 1	Pachymetry (Dependent variable)				0.691	0.478	0.008
	Area of fibrin glue	0.581	0.337	0.011	0.173		0.54
	IOP	0.681	0.464	0.002	0.554		0.063
POD 4	Pachymetry (Dependent variable)				0.652	0.425	0.016
	Area of fibrin glue	0.54	0.291	0.021	0.442		0.045
	IOP	0.257	0.066	0.303	0.379		0.081
POD 7	Pachymetry (Dependent variable)				0.856	0.732	0
	Area of fibrin glue	0.776	0.602	0	0.554		0.003
	IOP	0.713	0.509	0.001	0.423		0.017
POD 10	Pachymetry (Dependent variable)				0.8	0.64	0
	Area of fibrin glue	0.767	0.588	0	0.653		0.002
	IOP	0.547	0.299	0.019	0.254		0.164
POD 15	Pachymetry (Dependent variable)				0.613	0.376	0.029
	Area of fibrin glue	0.535	0.286	0.022	0.391		0.104
	IOP	0.502	0.252	0.034	0.333		0.162

Multivariate analysis of effects of mean area of fibrin glue and IOP on pachymetry, showing significant correlation between area of fibrin glue and pachymetry on POD 7, and POD 10, with IOP taken into consideration, p<0.05.

### Corneal specimens

#### Endothelial staining for live/dead cell assay

The viability of the corneal endothelium was tested using a live/dead cell assay, as described above. The vital stain revealed that the mean percentage of dead endothelial cells (±SEM) was 1.15% (±0.32) in the fibrin glue with normal IOP group, 1.50% (±0.43) in the fibrin glue with increased IOP group, and 1.18% (±0.38) in the control group. There was no significant difference between the percentages of dead corneal endothelial cells evaluated between the three groups (p>0.05). Therefore, intracameral injection of fibrin sealant did not have a significant effect on corneal endothelial cell viability compared to control eyes.

#### TUNEL assay

The TUNEL assay revealed that the mean percentages of TUNEL-positive epithelial cells (±SEM) were 0.15% (±0.06) in the fibrin glue with normal IOP group, 0.71% (±0.22) in the fibrin glue with increased IOP group, and 0.60% (±0.17) in the control group. The mean percentages of TUNEL-positive stromal cells (±SEM) were 0.21% (±3.26) in the fibrin glue with normal IOP group, 0.34% (±0.06) in the fibrin glue with increased IOP group, and 0.10% (±0.03) in the control group. The mean percentages of TUNEL-positive endothelial cells (±SEM) were 0.80% (±1.70) in the fibrin glue with normal IOP group, 0.69% (±0.09) in the fibrin glue with increased IOP group, and 0.58% (±0.21) in the control group. There was no significant difference between the number of TUNEL-positive cells in the epithelium, stroma, and endothelium between the three groups (p>0.05). Therefore, intracameral injection of fibrin sealant did not have a significant effect on apoptosis of the corneal epithelium, stroma, and endothelium compared to control eyes.

#### Electron microscopy (EM)–scanning EM and transmission EM

**Figure 6:**
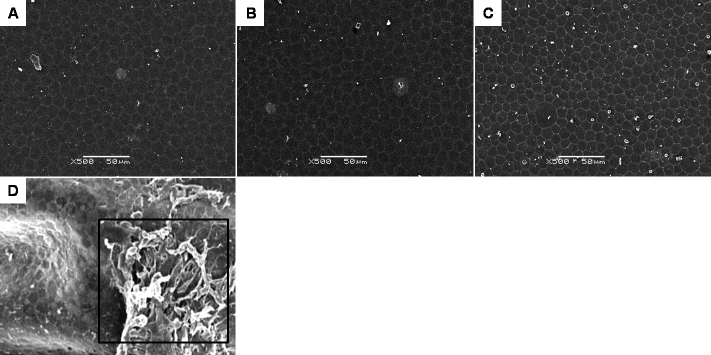
Scanning electron micrographs of rabbit corneal endothelium from the three different groups (**A**-**D**). All endothelial surfaces showed a regular hexagonal cellular mosaic and well-defined cell borders, with occasional pleomorphic cells. **D**: One corneal endothelium had residual TISSEEL glue attached to it (box), showing distortion to the surrounding endothelial cells. Magnification 500×; bar=50 µm.

**Figure 7:**
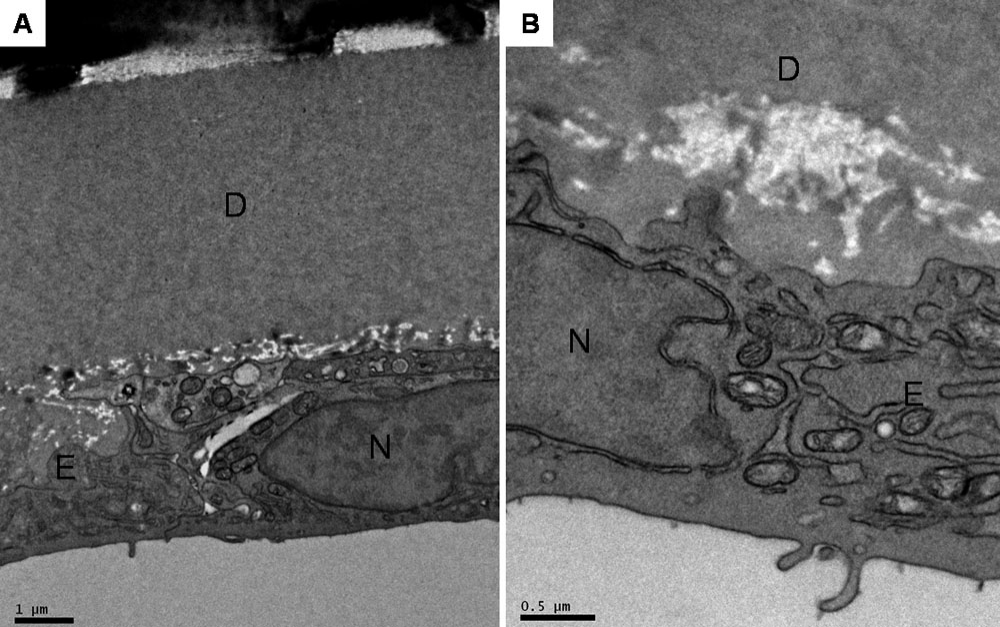
Transmission electron micrographs of a representative rabbit cornea (**A**, **B**) after the fibrin glue had dissolved, showing an endothelial cell (E) adherent to Descemet’s membrane (D), with a relatively regular cell surface and well-preserved intracellular organelles. The nucleus (N), rough endoplasmic reticulum, and mitochondria were seen in the cytoplasm. Of note, there was no mitotic response of the endothelial cells and no evidence of endothelial cell proliferation. Magnification: **A**, 7,100×; bar=1µm; **B**, 18,000×; bar=0.5µm.

The ultra-structure of the rabbits’ corneal endothelium was similar in all three groups and showed normal corneal endothelial morphology. The SEM of the endothelial surface showed a regular cellular hexagonal mosaic and well-defined and well-preserved cell borders. (Figure 6) Intercellular border thickness was normal. The TEM of the endothelial cells showed a relatively regular cell surface, with well-preserved intracellular organelles and intact zonula occludens. Of note, there was no mitotic response of the endothelial cells and no evidence of endothelial cell proliferation on TEM (Figure 7).

### Trabecular meshwork specimens

#### TUNEL assay

The TUNEL assay revealed that the mean percentages of TUNEL-positive cells (±SEM) were 0.32% (±0.20) in the fibrin glue with normal IOP group, 0.61% (±0.08) in the fibrin glue with increased IOP group, and 0.59% (±0.14) in the control group. There was no significant difference between the percentages of apoptotic trabecular meshwork cells between the three groups (p>0.05). Therefore, the intracameral injection of fibrin sealant did not have a significant effect on apoptosis of trabecular meshwork cells compared to control eyes.

#### Light microscopy and electron microscopy (EM)–transmission EM

**Figure 8:**
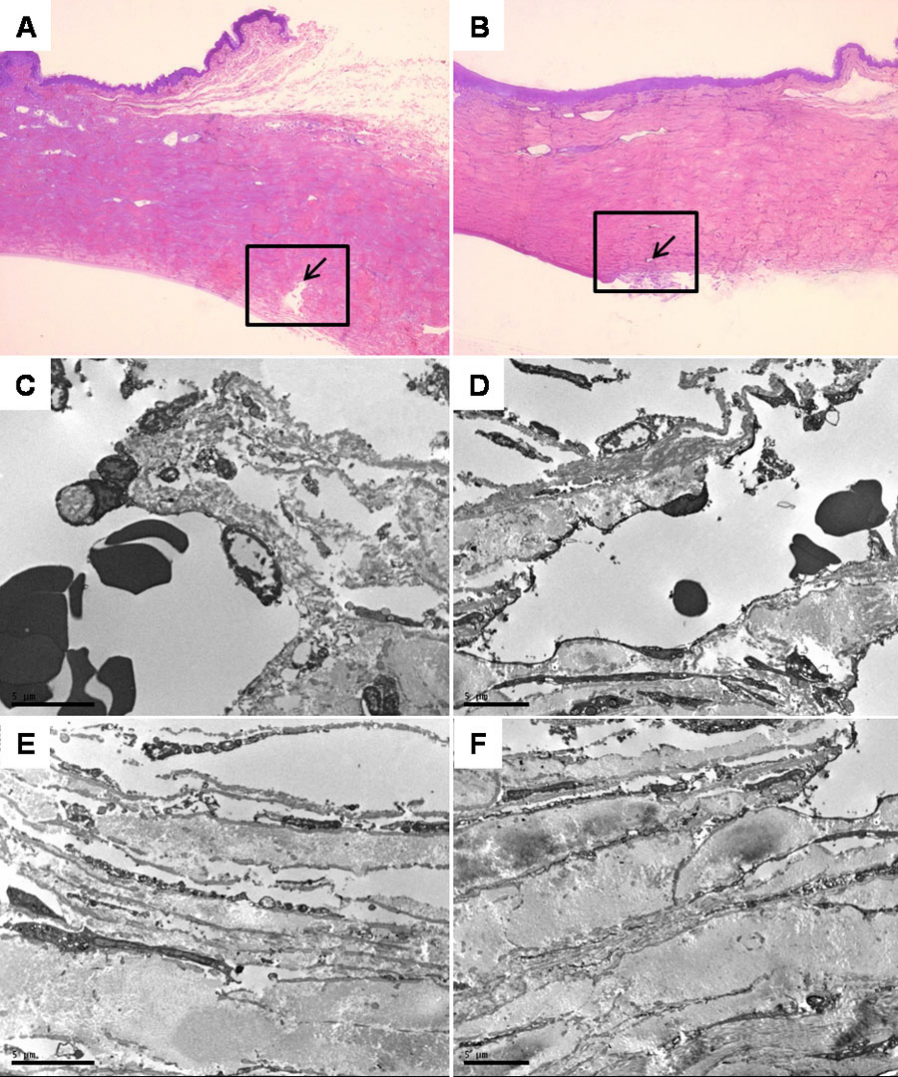
Light microscopy (**A**, **B**) and transmission microscopy (**C**-**F**) of the rabbits’ trabecular meshwork. Light micrographs of all groups showed the intact geometry of the meshwork. The trabecular meshwork (box) and Schlemm’s canal (arrow) was identified and isolated for TEM. A TEM of all groups showed a normal trabecular meshwork and Schlemm’s canal. The trabecular beams were characterized by a predominance of transversely sectioned normal collagen fibers in the central nucleus, widely separated by electron-lucent spaces. Elastin-like plaques varying in number and size were also observed in all three groups. These were randomly distributed in the central nucleus of the beams and under the basement membrane. Elongated, attenuated endothelial cells covered the outside of each beam. The inner wall of the Schlemm’s canal revealed giant vacuoles within the endothelial cells. There was no evidence of inflammatory cells present in the trabecular meshwork. Magnification: **A**, **B**; 100×; **C**, **D**; 2,200×; bar=5 µm; **E**, **F**; 2,800×; bar=5 µm.

Histology of the trabecular meshwork was similar in all three groups. The overall geometry of the meshwork was intact and the collagen beams retained a normal appearance (Figure 8). The TEM showed a normal trabecular meshwork and Schlemm’s canal in all three groups. The trabecular beams were characterized by a predominance of transversally sectioned normal collagen fibers in the central nucleus, widely separated by electron-lucent spaces. Elastin-like plaques varying in number and size were also observed in all three groups. These were randomly distributed in the central nucleus of the beams and under the basement membrane. Elongated, attenuated endothelial cells covered the outside of each beam. The inner wall of the Schlemm’s canal revealed giant vacuoles within the endothelial cells. There was no evidence of inflammatory cells in the trabecular meshwork (Figure 8).

### Iris specimens

#### Light microscopy

Light microscopy for all three groups demonstrated normal iris structures, showing the condensed layer of fibroblasts and melanocytes at the anterior border layer, with a loosely arranged matrix of extracellular matrix beneath, and two layers of pigment-containing epithelial cells lining the posterior iris surface (data not shown).

## Discussion

The aim of our study was to investigate the effect of intracameral injection of fibrin tissue sealant on the anterior segment structures of rabbit eyes. The results of this study showed that the quantity of intracameral fibrin sealant was significantly correlated with increased IOP and increased pachymetry in the early post-operative period. Complete dissolution of the fibrin sealant occurred between 15 and 30 days. Despite the increased IOP and pachymetry, live/dead cell and TUNEL assays showed no difference in endothelial cell viability in the cornea and a healthy trabecular meshwork regardless of post-operative course. Light and electron microscopy showed no abnormality in the corneal endothelium, trabecular meshwork, or iris architecture.

Our results showed that eyes injected with fibrin sealant had a significantly higher IOP compared to eyes injected with BSS. The increased IOP was controlled in one eye by topical medication alone and six out of seven eyes required therapeutic anterior chamber paracentesis in addition to topical anti-glaucoma treatment. We felt this was likely due to a trabecular meshwork blockage by fibrin sealant breakdown products, and in two eyes, an additional secondary angle closure, in which the small amount of injected fibrin sealant caused irido-corneal contact for the first few post-operative days. The amount of fibrin sealant correlated significantly with IOP on POD 1, following which the correlation ceased to be significant with the increased dissolution of the fibrin sealant over time. The lack of subsequent correlations was most likely due to the secondary interventions administered since the subsequent drop in IOP varied in different eyes. Hence, we advocate the judicious monitoring of IOP in the immediate post-operative period following the intracameral use of fibrin and avoiding direct injection to the drainage angle. The latter was more difficult in rabbit eyes because they have a shallower anterior chamber compared to human eyes. This was supported by our small pilot study in which the distribution of the fibrin glue applied was easier to control in human eyes [8].

Our results also showed that eyes injected with fibrin sealant had a significantly higher pachymetry compared to eyes injected with BSS. We speculate that the increased pachymetry was due to a combination of the raised IOP and the possible effect of the intracameral fibrin sealant on the physiologic function of the corneal endothelium. There was a significant correlation of central pachymetry with both increased IOP and the quantity of fibrin sealant (Table 3). This point is further illustrated by the fact that one rabbit eye had controlled IOP when topical anti-glaucoma medications were applied during much of the follow-up, and yet had a thickened cornea on ASOCT. Anterior segment optical coherence tomography scans from three other rabbits showed increased corneal thickness in areas in which the fibrin sealant was in contact with the endothelium and thinner corneal thickness in adjacent areas in which the endothelium was spared of fibrin sealant (data not shown). This seemed to suggest the possibility of fibrin sealant causing some temporary physiologic endothelial dysfunction by forming a physical barrier between the aqueous humor and the endothelial pumps, resulting in increased pachymetry. However, the endothelial dysfunction was only temporary since, following dissolution of the fibrin, the pachymetry returned to normal. The temporary corneal edema might have been partly due to the application of excessive glue in our case. We suspect that more surgically-controlled glue delivery with much thinner layers of glue may not have the same effect of inducing temporary corneal endothelial dysfunction. There was also no subsequent evidence of endothelial toxicity on live/ dead cell assays between fibrin sealant and control eyes. We aim to further examine this temporary endothelial dysfunction with in vitro tests using human research-grade eyes in a perfused artificial anterior chamber maintainer.

In all cases, the injected fibrin sealant within the AC was noted to be completely absorbed between post-operative days 15 and 30. Color slit-lamp photography is the conventional manner for assessing the AC, as in Figure 1. However, it is very difficult to quantify the amount of fibrin in the anterior chamber from a 2-D image. The novel use of ASOCT and Image J in this setting allowed a 3-D assessment of the fibrin in the eye. We found this method to be quantifiably reproducible (data not shown). Assessment of the rate of dissolution of the fibrin sealant was important since we were unsure whether any eyes needed additional use of r-TPA to speed up the dissolution. The results showed that the quantity of glue injected was significantly correlated with raised IOP in the immediate post-operative period and that the rate of dissolution of the fibrin from the anterior chamber was greater in those eyes that had more glue injected.

Despite the increased IOP and pachymetry, quantitative live/dead cell assays confirmed that sealant contact did not affect the viability of the corneal endothelial cells. The TUNEL assays showed no increase in apoptosis in the different layers of the cornea or the trabecular meshwork. Light and electron microscopy showed the preservation of the ultra-structures of the cornea and trabecular meshwork and of the architecture of the iris. Specifically, there were no inflammatory changes or mechanical damage from contact with the fibrin sealant noted on electron microscopy. The cytotoxicity of fibrin sealant has only been previously reported in in vitro cultures [9]. It has been shown to have minimal cytotoxicity in cultured bovine corneal epithelial, stromal, and endothelial cells. To our knowledge, there was no prior report evaluating the effect of intracameral fibrin sealant.

There were a few limitations in our study. One of the problems we encountered during the procedure was the difficulty in controlling the amount and thickness of fibrin sealant injected onto the endothelium and into the anterior chamber. This was mainly due to the fact that with the current injection system (the Duploject), the use of the fast setting glue often clogged the applicator; hence, delivering an exact amount with that system was not possible. That was why we used the mean area of intracameral fibrin sealant calculated to assess for correlations with raised IOP and pachymetry as opposed to the injected amount since that value would be too inaccurate. In all cases, excessive sealant inadvertently coated the iris, and in some cases, there was excessive sealant in the angle. That possibly led to the raised IOP in many of the cases and induced corneal edema. A more precise delivery device may obviate this problem. Unlike human endothelial cells, rabbit corneal endothelium maintains mitotic activity and regenerates after thermal and chemical insults [9]. However, the endothelial cells of rabbits take at least two weeks to replicate following injury, and only regain a normal appearance after four weeks [10]. We used a rabbit model since we predicted from our clinical pilot series that the speed of glue dissolution was shorter than the time for complete endothelial cell recovery, and for pragmatic reasons, including the previous reported use of the assessment of endothelial toxicity using rabbit models [11-13]. There were also no signs of endothelial mitotic activity seen in our electron microscopy results, hence supporting our conclusions. There exists the risk of viral transmission with the use of fibrin sealants. The sealer protein and thrombin components of TISSEEL are made from pooled human plasma [14]; hence, blood-borne viruses such as hepatitis B, hepatitis C, and human immunodeficiency virus could remain in commercial preparations even after viral reduction steps have been performed.

In summary, we demonstrated the intracameral use of fibrin glue in a rabbit model for the first time. The quantity of intracameral fibrin sealant was found to be significantly correlated with IOP and increased pachymetry in the early post-operative period. Complete dissolution of the fibrin sealant occurred between 15 and 30 days. Despite the increased IOP and pachymetry, there was no evidence of toxicity or structural damage to the corneal endothelium, trabecular meshwork, or iris. Further in vitro tests of fibrin sealant in human corneas are needed to verify our animal data.

## References

[1] Jackson MR Fibrin sealants in surgical practice: An overview. Am J Surg 2001 182 1S 7S 11566470 10.1016/s0002-9610(01)00770-x

[2] Hall RC Logan AJ Wells AP Comparison of fibrin glue with sutures for pterygium excision surgery with conjunctival autografts. Clin Experiment Ophthalmol 2009 37 584 9 19702708 10.1111/j.1442-9071.2009.02105.x

[3] O’Sullivan F Dalton R Rostron CK Fibrin glue: an alternative method of wound closure in glaucoma surgery. J Glaucoma 1996 5 367 70 8946291

[4] Asrani SG Wilensky JT Management of bleb leaks after glaucoma filtering surgery: use of autologous fibrin tissue glue as an alternative. Ophthalmology 1996 103 294 8 8594517 10.1016/s0161-6420(96)30701-x

[5] Sharma A Kaur R Kumar S Gupta P Pandav S Patnaik B Gupta A Fibrin glue versus N-butyl-2-cyanoacrylate in corneal perforations. Ophthalmology 2003 110 291 8 12578769 10.1016/S0161-6420(02)01558-0

[6] Duchesne B Tahi H Galand A Use of fibrin glue and amniotic membrane transplant in corneal perforation. Cornea 2001 20 230 2 11248838 10.1097/00003226-200103000-00027

[7] Kaufman HE Insler MS Ibrahim-Elzembely HA Kaufman SC Human fibrin tissue adhesive for sutureless lamellar keratoplasty and scleral patch adhesion. Ophthalmology 2003 110 2168 72 14597525 10.1016/S0161-6420(03)00832-7

[8] Por YM Tan YL Mehta JS Tan TH Intracameral fibrin tissue sealant as an adjunct in tectonic lamellar keratoplasty for large corneal perforations. Cornea 2009 28 451 5 19411966 10.1097/ICO.0b013e31818ad9d0

[9] Chen WL Lin CT Hsieh CY Tu IH Chen WY Hu FR Comparison of the bacteriostatic effects, corneal cytotoxicity, and the ability to seal corneal incisions among three different tissue adhesives. Cornea 2007 26 1228 34 18043181 10.1097/ICO.0b013e3181506129

[10] Van Horn DL Sendele DD Seideman S Buco PJ Regenerative capacity of the corneal endothelium in rabbit and cat. Invest Ophthalmol Vis Sci 1977 16 597 613 873721

[11] Kim T Holley GP Lee JH Broocker G Edelhauser HF The effects of intraocular lidocaine on the corneal endothelium. Ophthalmology 1998 105 125 30 9442788 10.1016/s0161-6420(98)91666-9

[12] Kim SY Park YH Lee YC Comparison of the effect of intracameral moxifloxacin, levofloxacin and cefazolin on rabbit corneal endothelial cells. Clin Experiment Ophthalmol 2008 36 367 70 18700925 10.1111/j.1442-9071.2008.01771.x

[13] Oh JY Wee WR Lee JH Kim MK Short-term effect of intracameral triamcinolone acetonide on corneal endothelium using the rabbit model. Eye 2007 21 812 8 16751761 10.1038/sj.eye.6702357

[14] Prescribing information on TISSEEL http://www.advancingbiosurgery.com/resources/pdfs/TISSEEL_PI_01.2010.pdf

